# Early intra-intensive care unit psychological intervention promotes recovery from post traumatic stress disorders, anxiety and depression symptoms in critically ill patients

**DOI:** 10.1186/cc10003

**Published:** 2011-01-27

**Authors:** Adriano Peris, Manuela Bonizzoli, Dario Iozzelli, Maria Luisa Migliaccio, Giovanni Zagli, Alberto Bacchereti, Marta Debolini, Elisetta Vannini, Massimo Solaro, Ilaria Balzi, Elisa Bendoni, Ilaria Bacchi, Valtere Giovannini, Laura Belloni

**Affiliations:** 1 Anaesthesia and Intensive Care Unit of Emergency Department, Careggi Teaching Hospital, Largo Brambilla 3, I-50139 Florence, Italy; 2 Regional Referral Center on Critical Human Relations, Careggi Teaching Hospital, Largo Brambilla 3, I-50139 Florence, Italy; 3 Medical Director of Careggi Teaching Hospital, Largo Brambilla 3, I-50139 Florence, Italy

## Abstract

**Introduction:**

Critically ill patients who require intensive care unit (ICU) treatment may experience psychological distress with increasing development of psychological disorders and related morbidity. Our aim was to determine whether intra-ICU clinical psychologist interventions decrease the prevalence of anxiety, depression and posttraumatic stress disorder (PTSD) after 12 months from ICU discharge.

**Methods:**

Our observational study included critical patients admitted before clinical psychologist intervention (control group) and patients who were involved in a clinical psychologist program (intervention group). The Hospital Anxiety and Depression Scale (HADS) and Impact of Event Scale-Revised questionnaires were used to assess the level of posttraumatic stress, anxiety and depression symptoms.

**Results:**

The control and intervention groups showed similar demographic and clinical characteristics. Patients in the intervention group showed lower rates of anxiety (8.9% vs. 17.4%) and depression (6.5% vs. 12.8%) than the control group on the basis of HADS scores, even if the differences were not statistically significant. High risk for PTSD was significantly lower in patients receiving early clinical psychologist support than in the control group (21.1% vs. 57%; *P *< 0.0001). The percentage of patients who needed psychiatric medications at 12 months was significantly higher in the control group than in the patient group (41.7% vs. 8.1%; *P *< 0.0001).

**Conclusions:**

Our results suggest that that early intra-ICU clinical psychologist intervention may help critically ill trauma patients recover from this stressful experience.

## Introduction

Several studies have reported that patients who need intensive care unit (ICU) treatment may experience psychological distress with increasing development of psychological illness and morbidity related to psychological disorders [1-4]. The presence of anxiety, depression and posttraumatic stress disorder (PTSD) symptoms have been reported in three studies to have increased by 40%, 30% and 60%, respectively, in ICU survivors [1,4-6].

The quality of life for critically ill patients after ICU treatment was found to be worst in patients who had undergone prolonged mechanical ventilation or had been admitted for severe trauma and sepsis [7]. Among critically ill patients, admission to the ICU as a result of major trauma may represent an additional risk factor because of the acutely stressful trauma experience. Studies which have followed ICU trauma patients after 1 year have reported a prevalence of PTSD symptoms of up to 30%, a prevalence up to 40% for anxiety and a prevalence up to 30% for depression [8-10]. Several factors (age and sex, duration of mechanical ventilation and ICU stay, unemployment, personality traits, factual and pain memory and educational status) have recently been associated with post-ICU psychological distress [4,11]. Recommendations available in the medical literature constitute only generic advisory statements on relational and psychological approaches to use with ICU patients without going into the mode, timing and characteristics of psychological intervention [12].

To improve the psychological outcome of ICU patients by helping the patients, their relatives and healthcare personnel elaborate the ICU experience, a Clinical Psychological Service was started in 2007 at the ICU of the Emergency Department of a tertiary referral center (Careggi Teaching Hospital, Florence, Italy). Given that, in our experience, clinical psychological activity is usually welcomed by patients and relatives, this study was carried out to verify that intra-ICU clinical psychological intervention can decrease the prevalence of anxiety, depression and PTSD symptoms in major trauma patients 1 year after discharge from the ICU.

## Materials and methods

### Patient selection and study design

This study was an observational study in which trauma patients admitted before the start of clinical psychologist intervention (January 2005 to March 2007) were included in the control group, and patients followed by clinical psychologists (April 2007 to August 2009) constituted the intervention group. All patients consecutively admitted to the ICU for major trauma from January 2005 to August 2009 were considered for the study. For each patient, data from institutional ICU and follow-up databases (FileMaker Pro; FileMaker, Inc., Santa Clara, CA, USA) and from the Italian Group for the Evaluation of Interventions in Intensive Care Medicine database (GiViTI Margherita Project; Istituto Mario Negri, Bergamo, Italy) were collected, including age, sex, body mass index (BMI), medical history (including psychiatric anamnesis), Glasgow Coma Scale (GCS) scores at admission and at ICU discharge, injury severity score (ISS), Abbreviated Injury Scale (AIS) score, Simplified Acute Physiology Score (SAPS) II, duration of mechanical ventilation, ICU and hospital length of stay (LOS), health status questionnaires (see "health status measurement" section), need for psychotherapy or psychiatric medications and timing of return to previous employment at 12 months. During the ICU stay, sedation was induced using propofol, fentanyl and/or midazolam infusions, depending on the patient's clinical condition. The 12-month follow-up sessions were conducted by properly trained nurses. This study includes procedures which were already integrated into the institutional follow-up protocol. The internal review board approved the study protocol, and informed consent for study participation and data publication was obtained.

Patients admitted during the study period were considered for enrollment on the basis of the following criteria: age between 18 and 75 years at admission, severe and/or critical injuries (ISS >15) [13], ICU LOS >72 hours, need for mechanical ventilation, ability to be interviewed during the ICU stay, completion of a follow-up examination at 12 months, absence of pre-existing psychiatric illness, absence of previous critical illness and absence of psychiatric medication use and/or any drug abuse or addiction in the patient's medical history.

Patients' psychiatric histories were collected by clinical psychologists and intensivists from the patients and/or the patients' relatives in collaboration with the family physician. Pre-existing psychiatric illness was excluded on the basis of the Diagnostic and Statistical Manual of Mental Disorders-Text Revision criteria.

### ICU organization and clinical psychologist intervention

The ICU of the Emergency Department at our hospital is a mixed ICU with 10 single-bed rooms. Nurse assistance is guaranteed at a variable ratio of one nurse for every two patients to one nurse for every patient as well as one to three health support operators per shift. The ICU is organized to permit a 24-hour stay in the ICU room for up to two next of kin or friends.

Patients (when actively collaborative) and/or relatives were informed about the Clinical Psychological Service at ICU admission. The psychological intervention program promoted by the ICU of the Emergency Department at Careggi Florence University Hospital is part of a project developed by Careggi Florence University Hospital and the Regional Referral Center on Critical Human Relations in cooperation with the Florence Health Society and Tuscany Region. The project started in April 2007 and concerns the prevention and treatment of the psychological impact of traumatic injury and critical illness in patients, caregivers and healthcare staff. The ICU has a staff of three clinical psychologists. Clinical psychologists are guaranteed to be on duty from 12:00 AM to 4:00 PM and are available through 24-hour on-call service. The annual cost of the Clinical Psychological Service is €30,000.

The phrase "psychological intervention in the ICU" covers a wide range of activities performed directly by clinical psychologists and a trained and supervised staff of intensivists and nurses, whose purpose is to provide emotional support and coping strategies to conscious patients with critical illness or major trauma injuries and their families.

The psychological interventions provided 24 hours per day include educational interventions, counseling and stress management approaches at the bedside, and they are documented in medical records. After recovery of consciousness, on average, patients receive five or six interventions from clinical psychologists during their ICU stay, including educational interventions, counseling, stress management, psychological support and coping strategies designed to ease the management of anxiety, depression, fear, hopelessness and helplessness and to reduce the discomfort produced by health conditions and medical procedures. The stress management intervention consists of cognitive and emotional restructuring. The interventions are also designed to help family members (starting during the phase when the patient is still unconscious) by promoting family-centred decision-making and supporting next of kin to choose appropriate interactions during their bedside visits. During the study period, family members were always met separately. All patients who underwent the psychological intervention were followed in the post-ICU wards after ICU discharge according to our institutional protocol.

### Health status measurement

The Impact of Event Scale-Revised (IES-R) questionnaire is one of the most often used self-report questionnaires for determining PTSD symptoms following trauma [14,15]. It consists of three subscales (eight items on intrusion, eight items on avoidance, and six items on hyperarousal) [16]. Each item is scored from 0 to 5. Scores of 33 or greater indicate a high probability of a PTSD diagnosis [17].

The Hospital Anxiety and Depression Scale (HADS) questionnaire consists of 14 items (seven items for anxiety and seven items for depression) [18]. Each item is scored on a scale ranging from 0 to 3, and a final score of 8 to 10 indicates a possible diagnosis of anxiety and/or depression; a score >11 confirms the diagnosis [4,19].

Quality of life was evaluated using the EQ5D™questionnaire [20]. The EQ5D™questionnaire consists of five items (Mobility, Self-Care, Usual Activities, Pain/Discomfort and Anxiety/Depression) scored from 1 to 3. Subjective perception of quality of life was estimated using the visual analogue scale (VAS), which is a 20-cm vertical visual analogue scale with the end points labeled best imaginable health at the top and worst imaginable health at the bottom with numeric values of 100 and 0, respectively.

### Statistical analysis

Statistical analyses were carried out using SPSS version 18 software (SPSS Inc., Chicago, IL, USA). Continuous variables were analyzed using a two-tailed Student's *t*-test or the Mann-Whitney *U *test as appropriate (D'Agostino and Pearson normality test). Categorical variables were examined using Fisher's exact test. A *P *value below 0.05 was considered an index of statistical significance. Continuous variables are expressed as means ± standard deviation (SD). Univariable comparisons were reported as odds ratios (ORs) with 95% confidence intervals (95% CIs).

A logistic regression model was adopted to investigate the predictors of anxiety, depression and PTSD symptoms in the overall population. Each predictor likely related to the outcome was evaluated according to statistical and clinical bases. Covariates associated with the response variables (*P *< 0.2) in univariate analysis, as well as those which could have a clinical meaning on the basis of the medical literature, were retained in the final model. Thus, the multivariable logistic regression analysis comprised age, gender, BMI, SAPS II, ISS, AIS score, GCS score at ICU admission and discharge, the presence of tracheostomy, the duration of mechanical ventilation and ICU LOS.

## Results

### General population

**Figure 1:**
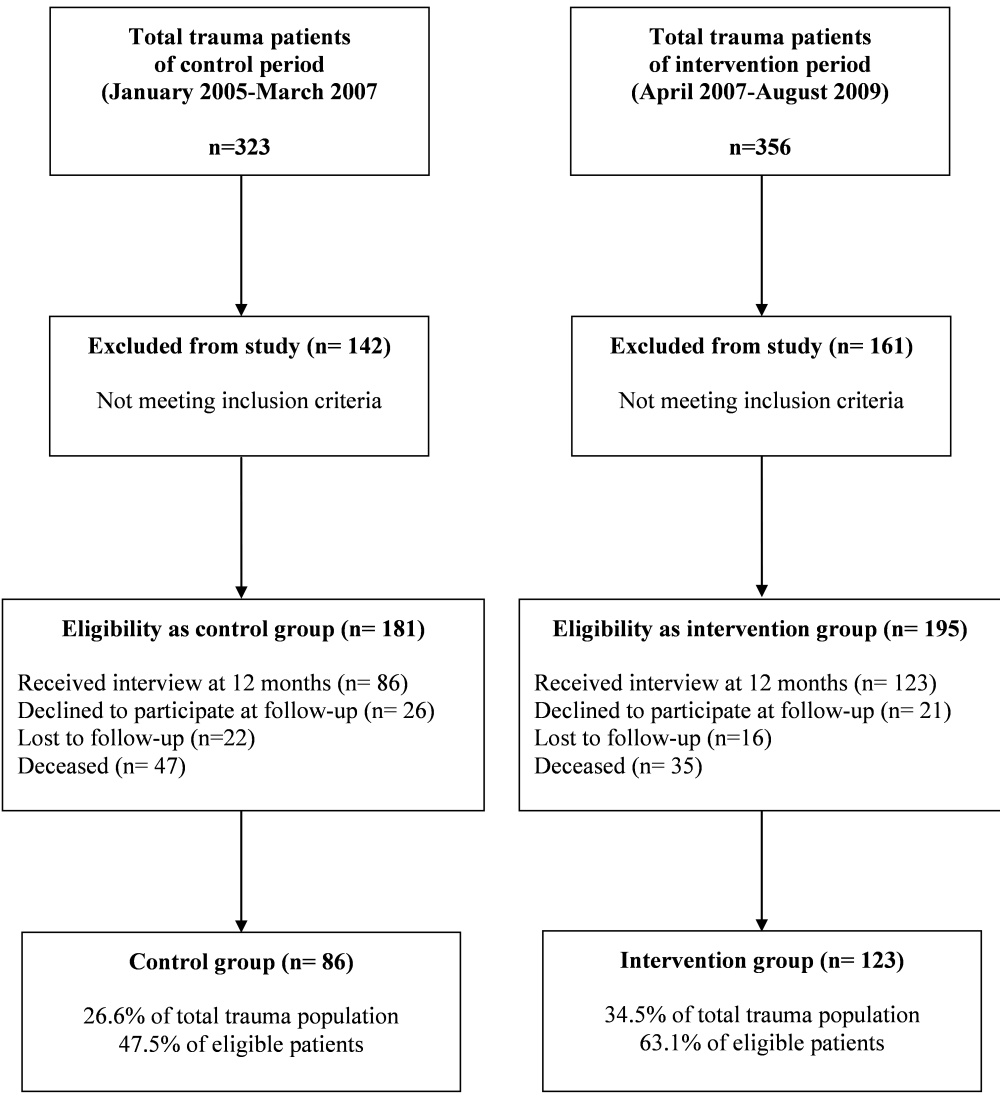
**Flow diagram of the study**.

Among 679 trauma patients admitted to the ICU during the whole study period, a total of 376 patients (55.4%) met the inclusion criteria as illustrated in the flow diagram shown in Figure 1. A total of 86 patients were enrolled in the control group, and 123 were enrolled in the intervention group. As summarized in Table 1, the groups were similar with regard to demographic and clinical characteristics.

**Table 1:** Comparison of baseline and clinical characteristics between control group and intervention group^a^

Characteristics	Control group (*n *= 86)	Psychologist group (*n *= 123)	*P *value
Age, yr (mean ± SD)	44.9 ± 19.8	43.7 ± 16.4	0.8212
Male sex, % (*n*)	72.1% (62)	83.7% (103)	0.0573
GCS score at admission, mean ± SD	9.0 ± 3.9	9.5 ± 4.2	0.6292
SAPS II, mean ± SD	38.5 ± 14.5	44.1 ± 20.5	0.2226
ISS, mean ± SD	28.9 ± 7.8	29.3 ± 9.1	0.3553
AIS score, mean ± SD			
Head and/or neck	3.1 ± 1.3	3.0 ± 1.4	0.4122
Face	2.1 ± 1.2	1.9 ± 1.4	0.1886
Chest	2.7 ± 1.3	2.5 ± 1.1	0.2212
Abdominal	1.8 ± 1.4	1.6 ± 1.6	0.3438
Extremity	2.4 ± 1.4	2.4 ± 1.5	0.7997
External	1.1 ± 0.8	0.9 ± 0.9	0.5651
Tracheostomy, % (*n*)	74.4% (64)	72.4% (89)	0.7541
Mechanical ventilation, days (mean ± SD)	14.2 ± 10.9	11.5 ± 9.9	0.1718
GCS at ICU discharge, mean ± SD	12.9 ± 2.9	13.6 ± 2.4	0.1395
ICU LOS, days (mean ± SD)	20.1 ± 11.3	17.8 ± 12.5	0.2738
Hospital LOS, days (mean ± SD)	39.2 ± 22.6	38.4 ± 24.5	0.3312

^a^Continuous data are expressed as means ± standard deviation (SD). Percentage data refer to the total population of each group. Statistical analysis was performed using the two-tailed Student's *t*-test and the two-tailed Fisher's exact test. *P *< 0.05 was considered a statistically significant result. AIS, Abbreviated Injury Scale; GCS, Glasgow Coma Scale; ICU, intensive care unit; ISS, injury severity score; LOS, length of stay; SAPS II, Simplified Acute Physiology Score II.

### Health status results

The diagnosis of anxiety and depression (categorical analysis for HADS scores >11) was lower in the clinical psychologist group than in the control group (8.9% vs. 17.4% and 6.5% vs. 12.8%, respectively) as confirmed by the Mann-Whitney *U *test (*P *= 0.0398 for anxiety and *P *= 0.0083 for depression). Despite the notable differences, the results were not statistically significant (Table 2). On the contrary, a high probability for a PTSD diagnosis was significantly lower in the clinical psychologist group than in the control group (21.1% vs. 57%; *P *< 0.001). On the IES-R Intrusion and Avoidance evaluation subscale, the scores were lower in the clinical psychologist group (Table 2).

**Table 2:** Comparison of test results between control group and intervention group^a^

Evaluation	Control group (*n *= 86)	Psychologist group (*n *= 123)	*P *value
HADS anxiety, % (*n*)	17.4% (15)	8.9% (11)	0.0879
HADS depression, % (*n*)	12.8% (11)	6.5% (8)	0.1448
IES-R subscores, mean ± SD			
Intrusion	11.3 ± 5.3	9.5 ± 4.4^b^	0.0255
Avoidance	12.1 ± 5.3	10 ± 3.4^b^	0.0152
Hyperarousal	8.7 ± 3.9	7.8 ± 2.7	0.0624
IES-R total score, mean ± SD	32.1 ± 14.2	27.2 ± 9.2^b^	0.0103
Posttraumatic stress disorder, % (*n*)	57% (49)	21.1% (26)^c^	< 0.0001
EQ5D™ subscores, mean ± SD			
Mobility	1.1 ± 0.4	1.4 ± 0.5^d^	0.0061
Self-care	1.1 ± 0.2	1.4 ± 0.5^c^	< 0.0001
Usual Activities	1.1 ± 0.3	1.5 ± 0.5^c^	< 0.0001
Pain/Discomfort	1.6 ± 0.5	1.6 ± 0.5	0.7580
Anxiety/Depression	1.3 ± 0.7	1.1 ± 0.3^b^	0.0257
EQ5D™ VAS, mean ± SD	72.4 ± 11.8	77.4 ± 9.1^b^	0.0495
Mental health interventions after hospital discharge, % (*n*)			
Psychotherapy	1.3% (1)	1.7% (2)	1.0000
Psychiatric medications	41.7% (36)	8.1% (10)^b^	< 0.0001
Return to previous employment at 12 months after hospital discharge, % (*n*)			
Within 3 months	14% (12)	23.6% (29)	0.1108
3 to 6 months	23.3% (20)	22.7% (28)	1.0000
6 to 12 months	22.1% (19)	17.9% (22)	0.4821

^a^Continuous data are expressed as means ± standard deviation (SD). Percentage data refer to the total population of each group. Statistical analysis was performed using Student's *t*-test, the Mann-Whitney *U *test and Fisher's exact test. P < 0.05 was considered statistically significant. ^b^*P *< 0.05; ^c^*P *< 0.001; ^d^*P *< 0.01. Both the Hospital Anxiety and Depression Scale (HADS) anxiety and depression diagnoses were made on the basis of a score >11. The posttraumatic stress disorder (PTSD) diagnosis was made on the basis of an Impact of Event Scale-Revised (IES-R) score >33. EQ5D™ [20] visual analogue scale (VAS) scores vary from 0 (worst imaginable health) to 100 (best imaginable health).

Subjective perception of quality of life on the basis of VAS evaluation was significantly higher in the clinical psychologist group than in the control group (77.4 ± 9.1 vs. 72.4 ± 11.8; *P *= 0.0495). Interestingly, the analysis of EQ5D™subscores showed that patients in the clinical psychologist group reported a significantly worse score than control group patients in the Mobility, Self-Care and Usual Activities components (Table 2). Despite these data, the subsequent self-evaluation of quality of life as measured by the EQ5D™VAS produced higher results.

The number of patients who needed anxiolytic and/or antidepressant therapy after hospital discharge were significantly greater in the control group than in the clinical psychologist group (41.7% vs. 8.1%), with an almost fourfold increased risk when adjusted for age and sex (OR, 3.79; 95% CI, 1.758 to 8.171; *P *< 0.001), whereas the results regarding time needed to return to previous employment at 12 months after hospital discharge were similar between the two groups (Table 2).

As shown in Table 3, patients at high risk for PTSD (IES-R scores ≥33) did not differ from patients with IES-R scores <33 with regard to demographic and clinical data, but the analysis showed that clinical psychologist intervention was strongly associated with the absence of PTSD-related symptoms (*P *< 0.001). The absence of psychological intervention was associated with a fivefold increased risk of PTSD development at 12 months (OR, 5.463; 95% CI, 2.946 to 10.13; *P *< 0.001). Among PTSD patients, 14 (19.2%) and 7 (9.6%) of them, respectively, had HADS scores >11 for anxiety and depression. Finally, the percentage of PTSD patients who required antidepressant therapy was significantly higher than non-PTSD patients (75.3% vs. 50.7%; *P *= 0.0006).

**Table 3:** Comparison of baseline and clinical characteristics between patients with and without a diagnosis of PTSD^a^

Characteristic	No high risk for PTSD (*n *= 136)	High risk for PTSD (*n *= 73)	*P *value
Age, yr (mean ± SD)	43.9 ± 19.1	44.9 ± 18.3	0.7554
Male sex, % (*n*)	76.5% (104)	76.7% (56)	1.0000
GCS score at admission (mean ± SD)	9.5 ± 4.1	8.9 ± 3.9	0.4771
SAPS II (mean ± SD)	42.2 ± 18.5	38.5 ± 14.6	0.2165
ISS (mean ± SD)	29.1 ± 5.8	28.8 ± 6.2	0.5596
AIS score (mean ± SD)			
Head/Neck	2.9 ± 1.3	3.2 ± 1.5	0.1655
Face	2.1 ± 1.1	2.2 ± 1.5	0.4105
Chest	2.6 ± 1.1	2.5 ± 1.6	0.7911
Abdominal	1.7 ± 1.2	1.7 ± 1.8	0.6086
Extremity	2.2 ± 1.4	2.3 ± 1.6	0.2143
External	0.7 ± 1.2	1 ± 1.3	0.1066
Tracheostomy, % (*n*)	69.9% (95)	76.7% (56)	0.1004
Mechanical ventilation, days (mean ± SD)	12.8 ± 9.9	13.9 ± 11.5	0.5695
GCS score at ICU discharge (mean ± SD)	13.3 ± 2.4	13.1 ± 3.0	0.6802
ICU LOS, days (mean ± SD)	18.6 ± 11.3	20.4 ± 12.3	0.3718
Hospital LOS, days (mean ± SD)	37.9 ± 22.8	40.4 ± 23.1	0.1192
Clinical psychologist intervention, % (*n*)	72.8% (99)^b^	32.9% (24)	< 0.001

^a^Continuous data are expressed as means ± standard deviation (SD). Percentage data refer to the total population of each group. Statistical analysis was performed using Student's *t*-test, the Mann-Whitney *U *test and Fisher's exact test. *P *< 0.05 was considered statistically significant. ^b^*P *< 0.001. AIS, Abbreviated Injury Scale; GCS, Glasgow Coma Scale; ICU, intensive care unit; ISS, Injury Severity Score; LOS, length of stay; PTSD, posttraumatic stress disorder; SAPS II, Simplified Acute Physiology Score II.

### Predictive factors for PTSD, anxiety and depression symptoms

Univariate analysis of the association between demographic and clinical variables and PTSD as well as anxiety and depression symptoms in the overall population is given in Table 4. As shown, no variables were clearly identified as independent predictors for PTSD, anxiety and depression development at 12 months after ICU discharge. The subsequent multivariate analysis model showed that predictors were a GCS score <9 at admission for PTSD symptoms and a GCS score <13 at discharge for anxiety symptoms. No significant predictors were found for depression symptoms.

**Table 4:** Univariate and multivariate analysis for anxiety, depression and PTSD symptoms in overall population^a^

	Univariate			Multivariate		
		
Variable	OR	95% CI	*P *value	OR	95% CI	*P *value
IES-R						
Age	0.995	0.988 to 1.022	0.190			
Sex	0.759	0.513 to 1.123	0.167			
GCS at admission	0.970	0.937 to 1.005	0.089	0.959	0.922 to 0.997	0.034
SAPS II	0.992	0.984 to 1.000	0.056			
ISS	0.891	0.833 to 1.014	0.151			
GCS at ICU discharge	0.979	0.954 to 1.004	0.104			
Anxiety						
Age	0.996	0.970 to 1.022	0.739			
Sex	1.121	0.342 to 3.672	0.851			
GCS at ICU discharge	0.892	0.758 to 1.049	0.166	0.841	0.704 to 1.003	0.054
Depression						
Age	1.020	0.989 to 1.051	0.206			
Sex	0.484	0.149 to 1.573	0.228			
GCS at admission	1.110	0.960 to 1.283	0.159			

^a^For clarity, only variables with *P *< 0.2 (univariate analysis) and *P *< 0.05 (multivariate analysis) are presented in the table. Age and sex are shown. GCS, Glasgow Coma Scale; ICU, intensive care unit; IES-R, Impact of Event Scale-Revised; ISS, injury severity score; LOS, length of stay; SAPS II, Simplified Acute Physiology Score II; OR, odds ratio; CI, confidence interval.

## Discussion

The main finding of this study is that, in a major trauma patient population, an early (intra-ICU) clinical psychologist intervention may have had a role in reducing the probability of a PTSD diagnosis at 12 months after discharge. A recent review [21] encourages psychological support of ICU patients by nurses, which was found to be associated with a better outcome (vital signs, decrease in pain ratings, anxiety, rate of complications, LOS, sleep improvement and patient satisfaction), but to our knowledge, no studies have directly quantified the effects of early clinical psychologist intervention in the ICU setting.

The symptoms of PTSD are clustered into three groups. The first two are specific to the traumatic etiology of the disorder: re-experience of the trauma and avoidance of stimuli likely to remind the patient of the trauma. Re-experience of the trauma includes intrusive memories and vivid images of the event during waking hours, which can be of such intensity that the person loses contact with their surroundings. Nightmares about the trauma are common. Avoidance of stimuli likely to remind the patient of the trauma include avoiding conversation, places, people and activities associated with the trauma. The third symptom group consists of hyperarousal (sleep disturbances, irritability and difficulty with concentration), and this cluster of symptoms commonly occurs in other psychological disorders as well as PTSD. The high-risk PTSD prevalence in our control group was higher (57%) than that recently reported by Toien *et al*. [11] (18%) in 118 trauma patients followed up at 12 months. This notable difference can be attributed to the different questionnaire used. In the present study, the IES-R was used, which includes the evaluation of hyperarousal, so that the total score is higher than on the IES, and the validated cut-off for the definition of high-risk PTSD patients remained a score of 33 [17]. In our sample, anxiety and depression prevalence at 12 months was notably (but not significantly) lower in the intervention group (Table 2). Since lack of significant results cannot authorize the conclusion regarding a beneficial effect of early clinical psychologist intervention, such differences encourage numerous further studies, also given that our statistics might be limited by the sample size.

In our total population, clinical predictors for IES and anxiety disorders were GCS score at admission and at ICU discharge, respectively, whereas no significant predictors were found for depression (Table 4). Previous studies identified several behavioral, social, personality traits and trauma- or ICU-related experiences as predictors for PTSD symptoms at 1 year post-ICU treatment [4,9,11]. In the present study, we cannot confirm what was previously reported because our primary interest was generally to assess the effects of early psychological intervention in a patient population affected by serious illness that arose acutely; this must be considered a limitation of the study. Also, despite the presence of the same internal standardized protocol for sedation in both groups, we cannot exclude the possibility that differences in sedative drug administration could have partially influenced the results. In the present study, we cannot show results concerning cognitive status: These data are lacking because that investigation of this feature started in 2010. Another limitation is the possible presence of pre-existing levels of depression and anxiety (not referred to during intensivist and clinical psychologist anamnesis collection). Moreover, potential data collection bias cannot be excluded. The interviewers were not aware of the study, but they were aware of the change in the ICU setting with the implementation of the Clinical Psychological Service. Finally, the difference in the percentage of patients who declined to participate at follow-up between the control and intervention groups (14.4% vs. 10.8%, respectively) (Figure 1), although comparable and not statistically significant, must be taken into consideration as a limiting factor. Also, the difference in mortality rates observed between patients eligible as controls (26%) and in the intervention group (18%) could have partially influenced the results of the study.

## Conclusions

Our data suggest that implementing ICU treatment with the presence of an intra-ICU clinical psychologist may help critically ill trauma patients recover from this acute, stressful experience. Although we await confirmation by further studies, since clinical psychologist intervention is not associated with any adverse effects, implementing this service should be considered in the ICU setting.

## Key messages

• Psychological disorders are frequent among ICU survivors.

• Early intra-ICU psychological intervention can decrease the risk of PTSD, anxiety and depression at 12 months after ICU discharge.

## Abbreviations

AIS: Abbreviated Injury Scale; GCS: Glasgow Coma Scale; HADS: Hospital Anxiety and Depression Scale; ICU: intensive care unit; ISS: injury severity score; IES-R: Impact of Event Scale-Revised; LOS: length of stay; PTSD: posttraumatic stress disorder; SAPS II: Simplified Acute Physiology Score II.

## Competing interests

The authors declare that they have no competing interests.

## Authors' contributions

AP, VG and LB organized the Clinical Psychological Service. AP, MB, MLM, DI and AB designed the study. AP, MB and GZ reviewed the literature. MLM, AB, MD, DI, EV, MS, IB, EB and IB collected the data. DI, AB and MD performed clinical psychologist interventions. MLM, AB, MD, DI, EV, MS, IB, EB and IB performed follow-up examinations. GZ performed statistical analysis. AP, DI, AB and GZ wrote the draft. All Authors revised the manuscript and approved the final version.

## References

[B1] Griffiths J Fortune G Barber V Young JD The prevalence of post traumatic stress disorder in survivors of ICU treatment: a systematic review Intensive Care Med 2007 33 1506 1518 10.1007/s00134-007-0730-z 17558490

[B2] Davydow DS Gifford JM Desai SV Needham DM Bienvenu OJ Posttraumatic stress disorder in general intensive care unit survivors: a systematic review Gen Hosp Psychiatry 2008 30 421 434 10.1016/j.genhosppsych.2008.05.006 18774425 PMC2572638

[B3] Rattray JE Hull AM Emotional outcome after intensive care: literature review J Adv Nurs 2008 64 2 13 10.1111/j.1365-2648.2008.04767.x 18721158

[B4] Myhren H Ekeberg O Toien K Karlsson S Stokland O Posttraumatic stress, anxiety and depression symptoms in patients during the first year post intensive care unit discharge Crit Care 2010 14 R14 10.1186/cc8870 20144193 PMC2875529

[B5] Scragg P Jones A Fauvel N Psychological problems following ICU treatment Anaesthesia 2001 56 9 14 10.1046/j.1365-2044.2001.01714.x 11167429

[B6] Eddleston JM White P Guthrie E Survival, morbidity, and quality of life after discharge from intensive care Crit Care Med 2000 28 2293 2299 10.1097/00003246-200007000-00018 10921555

[B7] Oeyen SG Vandijck DM Benoit DD Annemans L Decruyenaere JM Quality of life after intensive care: a systematic review of the literature Crit Care Med 2010 38 2386 2400 20838335 10.1097/CCM.0b013e3181f3dec5

[B8] Holbrook TL Hoyt DB Stein MB Sieber WJ Perceived threat to life predicts posttraumatic stress disorder after major trauma: risk factors and functional outcome J Trauma 2001 51 287 293 10.1097/00005373-200108000-00010 11493786

[B9] Ringdal M Plos K Lundberg D Johansson L Bergbom I Outcome after injury: memories, health-related quality of life, anxiety, and symptoms of depression after intensive care J Trauma 2009 66 1226 1233 10.1097/TA.0b013e318181b8e3 19088550

[B10] Schnyder U Moergeli H Trentz O Klaghofer R Buddeberg C Prediction of psychiatric morbidity in severely injured accident victims at one-year follow-up Am J Respir Crit Care Med 2001 164 653 656 11520732 10.1164/ajrccm.164.4.2008087

[B11] Toien K Myhren H Bredal IS Skogstad L Sandvik L Ekeberg O Psychological distress after severe trauma: a prospective 1-year follow-up study of a trauma intensive care unit population J Trauma 2010 69 1552 1559 10.1097/TA.0b013e3181e125f3 20664371

[B12] Davidson JE Powers K Hedayat KM Tieszen M Kon AA Shepard E Spuhler V Todres ID Levy M Barr J Ghandi R Hirsch G Armstrong D Clinical practice guidelines for support of the family in the patient-centered intensive care unit: American College of Critical Care Medicine Task Force 2004-2005 Crit Care Med 2007 35 605 622 10.1097/01.CCM.0000254067.14607.EB 17205007

[B13] Stevenson M Segui-Gomez M Lescohier I Di Scala C McDonald-Smith G An overview of the injury severity score and the new injury severity score Inj Prev 2001 7 10 13 10.1136/ip.7.1.10 11289527 PMC1730702

[B14] Elhai JD Gray MJ Kashdan TB Franklin CL Which instruments are most commonly used to assess traumatic event exposure and posttraumatic effects?: A survey of traumatic stress professionals J Trauma Stress 2005 18 541 545 10.1002/jts.20062 16281252

[B15] Boer KR van Ruler O van Emmerik AA Sprangers MA de Rooij SE Vroom MB de Borgie CA Boermeester MA Reitsma JB Dutch Peritonitis Study Group Factors associated with posttraumatic stress symptoms in a prospective cohort of patients after abdominal sepsis: a nomogram Intensive Care Med 2008 34 664 674 10.1007/s00134-007-0941-3 18197398 PMC2271079

[B16] Weiss DS Marmar CR Wilson JP, Keane TM The impact of event scale-revised Assessing Psychological Trauma and PTSD 1996 New York: Guilford 399 411

[B17] Creamer M Bell R Failla S Psychometric properties of the Impact of Event Scale-Revised Behav Res Ther 2003 41 1489 1496 10.1016/j.brat.2003.07.010 14705607

[B18] Zigmond AS Snaith RP The hospital anxiety and depression scale Acta Psychiatr Scand 1983 67 361 370 10.1111/j.1600-0447.1983.tb09716.x 6880820

[B19] Bjelland I Dahl AA Haug TT Neckelmann D The validity of the Hospital Anxiety and Depression Scale: an updated literature review J Psychosom Res 2002 52 69 77 10.1016/S0022-3999(01)00296-3 11832252

[B20] The EuroQol Group. EuroQol: a new facility for the measurement of health-related quality of life Health Policy 1990 16 199 208 10.1016/0168-8510(90)90421-9 10109801

[B21] Papathanassoglou ED Psychological support and outcomes for ICU patients Nurs Crit Care 2010 15 118 128 10.1111/j.1478-5153.2009.00383.x 20500650

